# Can Met-PET/CT Predict Sporadic Multiglandular Hyperparathyroidism? Report of a Case and Review of the Literature

**DOI:** 10.1155/2019/1791740

**Published:** 2019-05-15

**Authors:** Andreas Hillenbrand, Johannes Lemke, Doris Henne-Bruns, Ambros J. Beer, Vikas Prasad

**Affiliations:** ^1^Department of General and Visceral Surgery, University Medical Center Ulm, Ulm, Germany; ^2^Department of Nuclear Medicine, University Medical Center Ulm, Ulm, Germany

## Abstract

**Background:**

Primary hyperparathyroidism (pHPT) is a common endocrine disorder of the parathyroid glands. In most cases pHPT is caused by single gland disease, but about 10% of patients suffer from sporadic multiglandular disease (MGD). Patients with MGD, especially with ectopic parathyroid adenomas, have an increased risk for persistence/recurrence after surgery. Normally, sporadic MGD cannot be diagnosed preoperatively by parathyroid scintigraphy. We analyzed the potential of positron emission tomography/computed tomography (Met-PET/CT) to predict MGD.

**Methods and Case Presentation:**

We reviewed the literature, if preoperative Met-PET/CT could predict MGD in patients with pHPT. Further, we present a 71-year-old female with ectopic MGD. Preoperative localization via Met-PET/CT showed MGD with two areas suspected to be enlarged parathyroid glands (left lateral to the thyroid lobe and posterior mediastinum). Both diagnostic findings were extirpated and parathormone dropped into normal levels.

**Results:**

We identified four additional manuscripts, referring to MGD and Met-PET/CT with divergent results. Preoperative localization diagnostics using Met-PET/CT may not necessarily identify MGD. In most cases, Met-PET/CT localized only one adenoma and localizes larger adenomas more reliably than smaller adenomas.

**Conclusion:**

Identifying patients at risk of MGD preoperatively remains challenging. We found MET-PET/CT seems to predict MGD in patients with large size and high weight PTH adenomas. For ectopic parathyroid adenomas, accurate preoperative localization is the key to successful surgical removal. Met-PET/CT appears to have great potential in soft-tissue analysis of complex anatomical regions and can predict ectopic parathyroid adenomas.

## 1. Introduction

Primary hyperparathyroidism (pHPT) is a common endocrine disorder caused by parathyroid gland adenomas secreting excess amounts of parathyroid hormone (PTH). As a result, the blood calcium increases, which in turn causes kidney stones, psychiatric abnormalities, and/or bone disease. Several imaging modalities have been proposed for the detection of parathyroid adenomas in patients with primary hyperparathyroidism. Ultrasound (US) examination and parathyroid scintigraphy usually performed as SPECT with low-dose CT with Tc-99m methoxy-isobutyl-isonitrile (MIBI) are the predominant imaging techniques used in the setting of pHPT [1].

In patients with negative US or MIBI scans, C-11 methionine positron emission tomography/computed tomography (Met-PET/CT) might identify hyperfunctioning parathyroid glands [2]. Met-PET/CT is a highly sensitive method to localize parathyroid adenomas, particularly for cases in which conventional imaging techniques, such as ultrasound or sestamibi scintigraphy, have failed [3].

Therapeutically, surgical resection of the hormone-releasing adenoma(s) remains standard therapy. Most patients with pHPT have a single gland adenoma. However, about 10% of patients suffer from sporadic multiglandular disease (MGD) [4]. Identifying patients at risk of MGD preoperatively remains challenging. Patients with MGD are considered to have higher risk of persistent disease. Therefore, a possible identification of patients with MGD could reduce the risk of a postoperative persistent hyperparathyroidism [5]. Further, ectopic parathyroid glands are found in about 1–3% of the population [6]. For ectopic parathyroid adenomas, accurate preoperative localization is the key to successful surgical removal. Met-PET/CT appears to have great potential in soft-tissue analysis of complex anatomical regions [7]. Aim of this study was to analyze if Met-PET/CT can predict MGD.

## 2. Case Report

Here, we report a case of a 71-year-old female diagnosed with MGD. The patient had no concurrent diseases and was not under any long-term medication. During a routine checkup, a hypercalcemia (3.7 mmol/l) with elevated parathyroid hormone (PTH: 233 pg/ml) was detected. Despite the very high serum calcium levels, the patient did not suffer from pHPT-specific symptoms such as kidney stones. A bone density measurement was not done. A preoperative neck and thyroid ultra-sonogram and a 99mTc-sestamibi SPECT/CT did not reveal an enlarged parathyroid adenoma in any location. An ultra-sonogram revealed a slightly enlarged multinodular thyroid and 99mTc-sestamibi SPECT/CT an uptake of radiotracers in the left lower thyroid, which intraoperatively turned out to be an inflammatory thyroid node.

**Figure 1:**
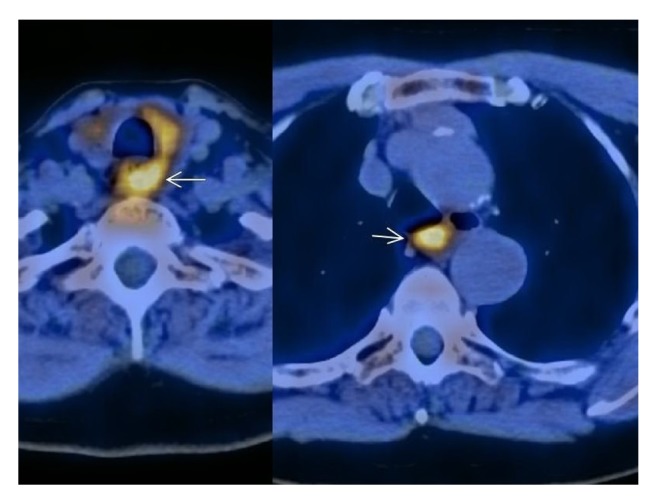
Preoperative localization via Met-PET/CT showed an intense uptake left lateral to the thyroid lobe and in the posterior mediastinum.

**Figure 2:**
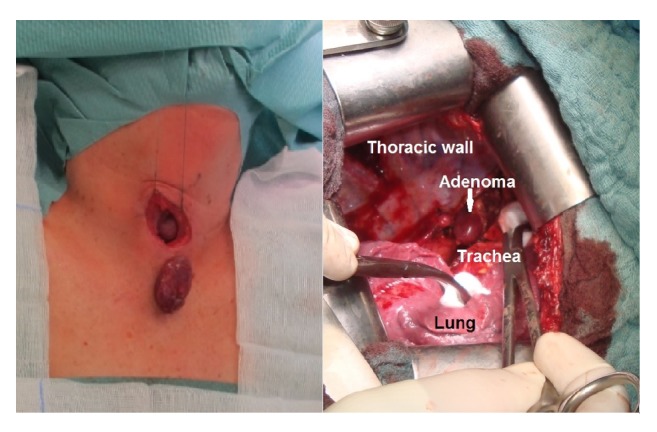
Extirpation of the left upper parathyroid gland and left thoracotomy with enlarged mediastinal parathyroid adenoma.

Preoperative localization via Met-PET/CT showed a MGD with two areas suspected to be enlarged parathyroid glands (left lateral to the thyroid lobe and posterior mediastinum; Figure 1). Both diagnostic findings were extirpated in a two-stage procedure. First, a Kocher transverse collar incision was performed and the left thyroid area was explored. An enlarged left upper parathyroid gland (weight: 7.6 g, size: 4 × 2.5 × 1 cm) was extirpated, and a left caudal parathyroid gland was intraoperatively found to be normal in size (Figure 2). Further, a lower pole resection of the left thyroid (histology: inflammatory goiter node) and a left-sided thymus resection (histology: atrophic thymus with a cyst) was performed. Intraoperative PTH levels declined from 342 pg/ml to 197 pg/ml 10 minutes after extirpation. Three months later, a right thoracotomy was performed, and an enlarged parathyroid gland was extirpated from the posterior mediastinum (weight: 10.2 g size: 5 × 2.3 × 1.5 cm; Figure 2). Afterwards, intraoperative PTH levels declined from 189 pg/ml to 43 pg/ml at 10 minutes after extirpation. The postoperative course was uneventful and patient was discharged after one week with normal serum calcium levels (2.3 mmol/l; PTH: 51 pg/ml).

## 3. Discussion

The current imaging standard for patients with hyperparathyroidism is MIBI scintigraphy in combination with ultrasonography [1]. The sensitivity and specificity of these methods are high (approximately 90%) in patients with solitary parathyroid adenomas, but low if multiple gland pathology is encountered [8]. Normally, sporadic MGD cannot be diagnosed preoperatively by parathyroid scintigraphy due to low accuracy, sensitivity and specificity of any preoperative localization tests performed, although two cases of double parathyroid adenoma evidenced by 99mTc-MIBI SPECT/CT are reported [5, 9–11]. Moreover, negative preoperative localization studies are even highly predictive of MGD [4]. In our reported case, MGD was preoperatively diagnosed by Met-PET/CT.

To identify additional cases of double adenomas identified preoperatively by Met-PET/CT, a Medline search was performed using the terms “Met-PET/CT”, “parathyroid adenoma”, and “double adenoma”. Publications were screened and manuscripts relevant for the current study were selected for further analysis. We identified four additional manuscripts, referring to MGD and Met-PET/CT. Interestingly, we found divergent results in these reports. In the first three case series, six patients with MGD and a preoperative Met-PET/CT were reported. In only one of these patients Met-PET/CT localized more than one hyperfunctioning parathyroid gland [12–14]. Weber et al. analyzed 2017 results of preoperative Met-PET/CT in patients with negative Tc-99 sestamibi (MIBI) scintigraphy. Three patients with double adenomas were reported and Met-PET/CT correctly located five of six parathyroids [2].

Review of the literature revealed that preoperative localization diagnostics using Met-PET/CT may not necessarily identify MGD. In the majority of cases, Met-PET/CT localized only one adenoma in these cases, comparable to MIBI scintigraphy.

It appears that Met-PET/CT localizes larger adenomas more reliably than smaller adenomas. Braeuning et al. reported in their case series that all lesions missed by PET/CT had a size smaller than 9 mm and a volume of less than 0.2 ml [12]. Moreover, Weber et al. reported a highly significant correlation between true-positive findings and size of parathyroid adenomas. Reported mean size of adenomas was 1.81 ± 0.84 cm and mean weight was 1.50 ± 2.56 g. Weight and size of the two parathyroid glands in our patient were comparably very high (7.6 and 10.2 g or 4 × 2.5 × 1 cm and 5 × 2.3 × 1.5 cm). The large size and high weight are potentially the reason why both adenomas in our case were detected in the preoperative Met-PET/CT. As mentioned before, some studies have suggested that MIBI scintigraphy also has the potential to identify MGD. However, whether Met-PET/CT identifies MGD more reliably than MIBI scintigraphy remains to be shown in future studies.

Therapy of patients with MGD is challenging since there is no reliable diagnostic tool to identify patients with MGD preoperatively. In particular, ectopic adenomas in patients with MGD are difficult to detect. Our observation suggests that the use of Met-PET/CT can help to identify MGD preoperatively or prior to second intervention. In patients with larger adenomas, Met-PET/CT is able to predict MGD.

## Data Availability

Data and the patient's records can be found in the hospital's documentation system.
